# Co-delivery of EGCG and melittin with self-assembled fluoro-nanoparticles for enhanced cancer therapy

**DOI:** 10.18632/aging.204769

**Published:** 2023-06-05

**Authors:** Meiling Sun, Yue Wu, Zheyu Zhou, Siyuan Liu, Shuai Mao, Guoqiang Li

**Affiliations:** 1 Department of Hepatobiliary Surgery, Affiliate Drum Tower Hospital, Medical School of Nanjing University, Nanjing 210008, Jiangsu Province, P.R. China; 2 Department of General Surgery, Nanjing Drum Tower Hospital, Chinese Academy of Medical Science and Peking Union Medical College, Graduate School of Peking Union Medical College Nanjing, Nanjing 210008, Jiangsu Province, P.R. China; 3 Department of Hepatobiliary Surgery, Nanjing Medical University Nanjing, Nanjing 211166, Jiangsu Province, P.R. China

**Keywords:** PD-L1, FEGCG, EGCG, melittin, fluorine

## Abstract

Purpose: Melittin (MPI) is a potential anticancer peptide due to its abilities of antitumor and immunomodulatory functions. Epigallocatechin-3-Ogallate (EGCG), a major extract of green tea, has shown great affinity for various types of biological molecules, especially for peptide/protein drugs. The aim of this study is to prepare a fluoro- nanoparticle (NP) formed by self-assembly of fluorinated EGCG (FEGCG) and MPI, and evaluate the effect of fluorine modification on MPI delivery and their synergistic antitumor effect.

Methods: Characterization of FEGCG@MPI NPs was determined by dynamic light scattering (DLS) and transmission electron microscope (TEM). Biology functions of FEGCG@MPI NPs were detected by hemolysis effect, cytotoxicity, apoptosis, cellular uptake with confocal microscopy and flow cytometry. The protein expression levels of Bcl-2/Bax, IRF, STATT-1, P-STAT-1, and PD-L1 were determined via western blotting. A transwell assay and wound healing assay were used to detect the cell migration and invasion. The antitumor efficacy of FEGCG@MPI NPs was demonstrated in a subcutaneous tumor model.

Results: Fluoro-nanoparticles could be formed by self-assembly of FEGCG and MPI, and fluorine modification on EGCG could ameliorate the side effect and delivery of MPI. The promoted therapeutics of FEGCG@MPI NPs could be achieved by regulating PD-L1 and apoptosis signaling, which might involve pathways of IRF, STAT-1/pSTAT-1, PD-L1, Bcl-2, and Bax *in vitro*. Moreover, FEGCG@MPI NPs could significantly inhibit the growth of tumor *in vivo*.

Conclusions: FEGCG@MPI NPs may offer a potential platform and promising strategy in cancer therapy.

## INTRODUCTION

Hepatocellular carcinoma is one of the main causes of death throughout the world. Compared with traditional therapeutics [1, 2] (i.e., chemotherapy, radiotherapy, and surgical treatment), peptide-based therapeutic strategies may provide more choices for its clinical application, including diagnosis, prognosis, and treatment [3–5]. Peptides can recognize and bind to the specially expressed proteins on tumor cells to guide cell killing with multiple favorite properties [6]. Recently, melittin (MPI), a cytolytic peptide derived from bee venom with 26 amino acids (GIGAVLKVLTTGLPALIS WIKRKRQQ), has attracted new insights in cancer therapy due to its abilities of antitumor and immunological regulation [7–9]. However, some dilemmas like hemolysis and toxicity still limit its clinical use. Therefore, there is an urgent need to develop novel strategies to ameliorate its anti-tumor efficacy as well as reduce its side effects.

A range of delivery strategies has been explored to overcome the above dilemmas of MPI, including nanomaterial-based delivery, hydrogel-based delivery, genetic engineering, living cell-based delivery, and protein fusion strategies [10–14]. Despite the explosive growth of research on MPI-based delivery system, the translation of these platforms is far from research to clinic since the carrier is the excess excipients that may bring unnecessary immunogenicity and toxicity to the whole system [15]. One potential solution is to select the carrier with safety and therapeutics. (–)-epigallocatechin-3-Ogallate (EGCG), a major extract of green tea, has shown great safety in many clinical trials, and various biological functions, such as anti-inflammatory, anticancer, antimicrobial, and immunomodulation effects [16–18]. Specifically, EGCG is a potential programmed death-ligand 1 (PD-L1) inhibitor that can regulate the response of tumor cells to immunotherapy [19, 20]. More appealingly, EGCG exhibits strong binding affinity with a great variety of biomolecules, such as nucleic acids and proteins/peptides [21]. The fabricated EGCG/biomolecule nanoparticles (NPs) achieved high delivery efficiency and minimal toxicity. The inspiration of these studies reinforced the idea that the combination of EGCG and MPI represents a novel strategy to improve the anti-tumor effects and decrease the side effects.

Here, we first functionalized EGCG with fluorinated bridges to obtain fluorinated EGCG (FEGCG) since the lipophobic and hydrophobic feature of fluorine atoms is beneficial for biomolecules delivery [22, 23]. Furthermore, FEGCG@MPI NPs were fabricated via hydrogen-bond interactions. As a novel system, FEGCG@MPI NPs can not only decrease PD-L1 expression to break the programmed death-1 (PD-1)/PD-L1 interaction but also induce apoptosis of tumor cells. These combined regulations can augment the cancer immunotherapy of MPI. Such a novel system of FEGCG@MPI NPs, with its efficacy demonstrated in the hepatoma model, will serve as a promising platform for MPI-based cancer therapies.

## RESULTS

### Preparation and characterization of FEGCG@MPI NPs

**Figure 1:**
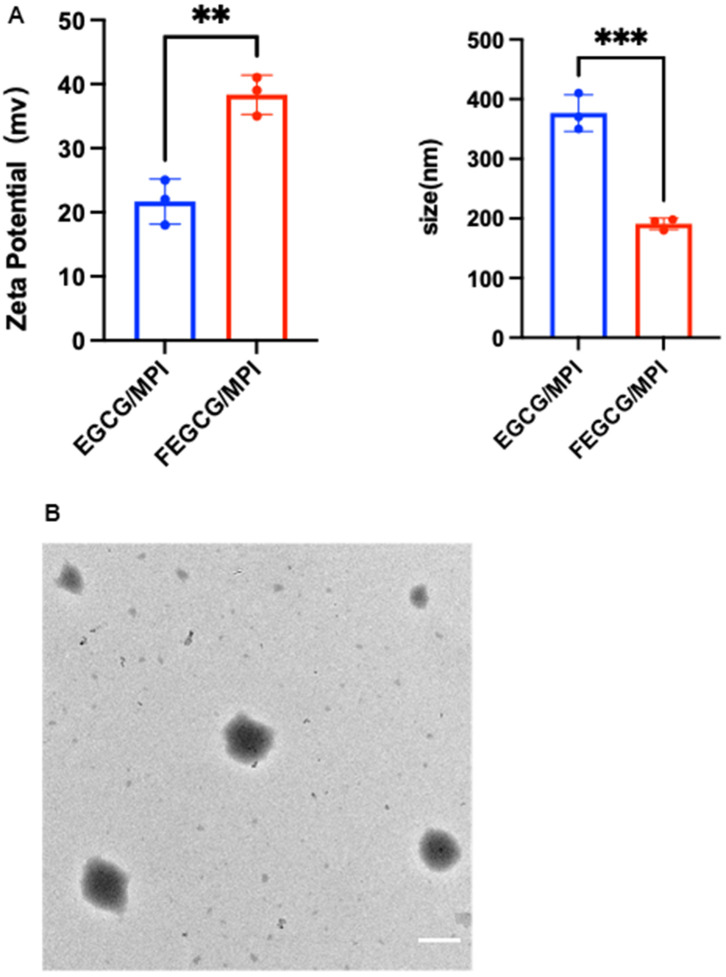
**Characterization of FEGCG@MPI NPs.** (**A**) Size contribution of FEGCG@MPI NPs, and EGCG@MPI NPs. Zeta potential of FEGCG@MPI NPs, and EGCG@MPI NPs. (**B**) TEM images of FEGCG@MPI NPs. Scale bar = 100nm.

FEGCG and MPI are bonded by hydrogen bond. As expected, the NPs could be self-assembled. FEGCG/MPI or EGCG/MPI, and their particle size was about 299, 499 nm, Zeta potential was 21, 38 mv (Figure 1A). Transmission electron microscope (TEM) results confirmed the successful formation of FEGCG/MPI (Figure 1B). These results demonstrated that FEGCG/MPI system has the potential to deliver biomolecules ranging from small molecule drugs to larger macromolecules.

### Cytotoxicity, apoptosis and cellular uptake of FEGCG@MPI NPs

Haberman and Zhan qi obtain melittin from bee venom. Because of its strong toxicity (mainly hemolytic activity), its clinical application is limited. It is generally that the hemolytic toxins of melittin are mainly based on the transmission control mechanism. Melittin can directly form holes in the biofilm to cause hemolysis, and can also be degraded by phospholipase on the activated red cell membrane. We guess FEGCG@MPI NPs can reduce the hemolytic reaction of MPI, which may be due to the formation of nanoparticles that encapsulate phospholipids to avoid the attack of MPI perforation. Hemolytic activity assay indicated that (100μg/mLFEGCG+3μg/mL MPI) mixed administration group had less hemolytic reaction than free MPI (Supplementary Figure 1).

**Figure 2:**
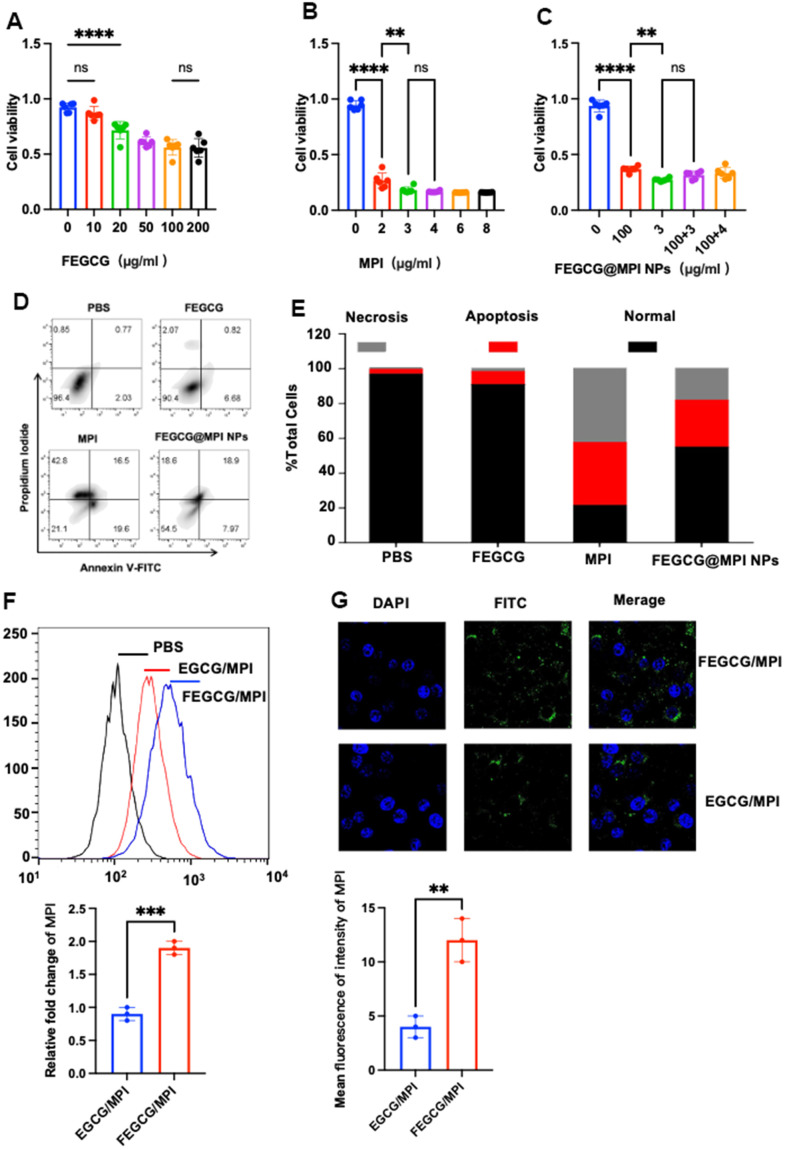
**Biology functions of FEGCG@MPI NPs *in vitro*.** (**A**–**C**) CCK-8 assays were used to detect the viability of Hepatoma cells (Hep 3B cell) after treatment with various concentrations of FEGCG, MPI and FEGCG@MPI NPs separately. (**D**, **E**) Apoptosis of Hep3B cells after PBS, FEGCG, MPI, and FEGCG/MPI treatments (*n* = 4). (**F**, **G**) The cytosolic delivery efficiency of EGCG/MPI, and FEGCG/MPI complexes determined by confocal microscopy and flow cytometry. Scale bar=100μm. Data are presented as the mean ± standard deviation. *P < 0.05, **P < 0.01 by an unpaired two-tailed Student’s t-test (two groups) or one-way analysis of variance (ANOVA) with Turkey’s multiple comparisons (three groups).

For the exploration of the activity of FEGCG, MPI and its synergy on the growth of Hepatoma cells, CCK-8 assays were used to detect the viability of Hepatoma cell (Hep 3B cell) after treatment with various concentrations of FEGCG, MPI and FEGCG@MPI NPs separately. As presented in (Figure 2A, 2C), FEGCG inhibited the growth of Hep3B cell in a dose-dependent manner. Up to 100μg/mL of FEGCG shows significant decrease in viability in Hep3B cell. In addition, melittin also shows a significant inhibitory effect on Hep3B cell after incubation for 24 h. As shown in (Figure 2B), cell activity was significantly reduced when it was treated with MPI. However, the concentration was 3μg/mL, cell activity no longer changes with concentration. Finally, to explore the effect on cell activity after preparing nanoparticles by mixing two single drugs, which is shown in (Figure 2C), a mixture of 100μg/mL FEGCG and 3μg/mL MPI was added to Hep3B cells, killing effect on tumor cells is the most obvious (Figure 2C) and apoptosis peaks were significantly increased (Figure 2D, 2E).

Confocal microscopy and flow cytometry experiments demonstrated that FEGCG/MPI achieved the highest cell uptake. It is further confirmed that FEGCG@MPI NPs has better efficiency in Hep3B cells (liver cancer) (Figure 2F). These results indicated that the FEGCG/MPI was a feasible and effective strategy to overcome the limitations in biomolecule delivery (Figure 2F, 2G).

### Regulation of PD-L1 and apoptosis of FEGCG@MPI NPs *in vitro*

**Figure 3:**
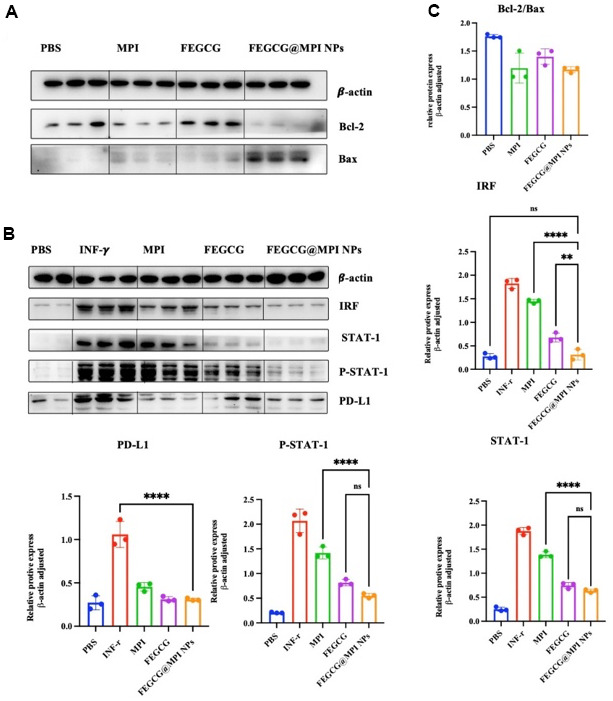
**Effect of FEGCG@MPI NPs on apoptosis and PD-L1 pathways.** (**A**) Both MPI and FEGCG could reduce the ratio of Bcl-2/Bax, and the combined effect was better than MPI and FEGCG alone. (**B**) The results showed that the protein contents of IRF, STATT-1, P-STAT-1 and PD-L1 in FEGCG@MPI NPs group were significantly decreased. (**C**) The experiment was performed for three times. Data are presented as the mean ± standard deviation. *P < 0.05, **P < 0.01 by an unpaired two-tailed Student’s t-test (two groups) or one-way analysis of variance (ANOVA) with Turkey’s multiple comparisons (three groups).

Bcl-2 family proteins are major regulators of apoptosis in health and disease. In the present study, we evaluated the expression of Bax and Bcl-2 genes in hepatocellular carcinoma to investigate the regulatory effect of FEGCG and MPI on the Bcl-2 family. Bcl-2 protein family is a special family. 25 homologous proteins of Bcl-2 family have been found. Bad and Bax promote apoptosis, while Bcl-2 and Bcl-2 inhibit apoptosis. It can be seen from (Figure 3A) that FEGCG can regulate the increase of Bax and the decrease of Bcl-2, and melittin can significantly reduce the protein content of Bcl-2. From the study, it can be concluded that mixed administration can significantly reduce the protein content of Bcl-2 and increase the protein content of Bad. Programmed death ligand 1 (PD-L1) is the ligand of programmed death 1 (PD-1) and an important immune checkpoint protein, which is responsible for negatively regulating the stability and integrity of T cell immune function. However, the response rate of patients to current anti PD-1 or anti PD-L1 treatment is still very low, and many initial responders eventually develop drug resistance to these treatments. FEGCG, the raw material of this study, can reduce the content of PD-L1 through transcriptional level, thereby effectively inhibiting the growth of liver cancer, thereby reducing the expression of PD-L1. At the same time, it can be seen from (Figure 3B) that mixed administration can enhance the effect of FEGCG in reducing PD-L1, and cooperate with MPI to play an anti-tumor role.

### Migration and invasion abilities of FEGCG@MPI NPs *in vitro*

**Figure 4:**
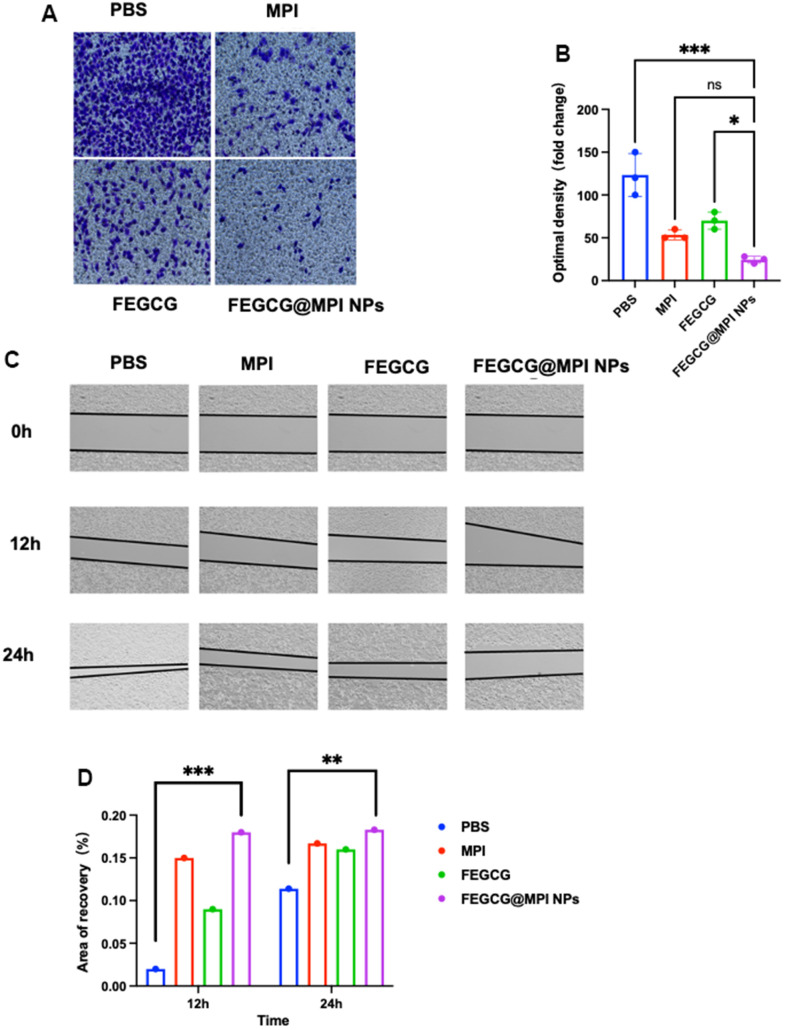
**Effect of FEGCG@MPI NPs on invasion and migration of Hep 3B cells.** (**A**) Invasion of Hep3B cells treated with PBS, MPI, FEGCG, FEGCG@MPI NPs. The FEGCG@MPI NPs group had significantly less cell migration than the PBS, MPI and FEGCG groups. (**B**) Data are presented as the mean ± standard deviation. *P < 0.05 vs MPI or FEGCG, **P < 0.01 vs MPI or FEGCG. (**C**) Scratch of Hep3B cells treated with PBS, FEGCG, MPI, FEGCG@MPI NPs for 0, 12 and 24 hours respectively. (**D**) Data are presented as the mean ± standard deviation. *P < 0.05, **P < 0.01 by an unpaired two-tailed Student's t-test (two groups) or one-way analysis of variance (ANOVA) with Turkey's multiple comparisons (three groups).

Cancer cell invasion refers to local invasion or distant metastasis of tumor cells. In 1889, Paget put forward the theory of “seed and soil”, which believed that only when appropriate tumor cells (seeds) had special affinity with the growth environment provided by specific tissues or organs (soil), they would transfer. In recent years, there has been a new understanding of this theory. In 1977, Fidler et al. first confirmed the heterogeneity of tumor cells, that is, tumor cells in primary tumor tissues have different biological characteristics, including some cells with metastatic potential. Under the selective pressure of the host, these cells have the ability to migrate and infiltrate, form tumor thrombi, survive in the circulation, stay in the distant capillary bed, infiltrate the organ parenchyma and proliferate, and finally form a monoclonal metastasis. The main cause of death in HCC patients is the invasion and migration of cancer cells. MPI can well inhibit the invasion of liver cancer cells. This article mainly explored whether FEGCG@MPI NPs can reduce more the invasion and migration of tumor cells. FEGCG@MPI NPs significantly inhibited cell migration (Figure 4A, 4B) and decreased wound healing (Figure 4C, 4D) compared with other control cells. These results indicated that compared with MPI and FEGCG, FEGCG@MPI NPs can significantly reduce cell invasion and migration.

### Antitumor efficacy of FEGCG@MPI NPs *in vivo*

**Figure 5:**
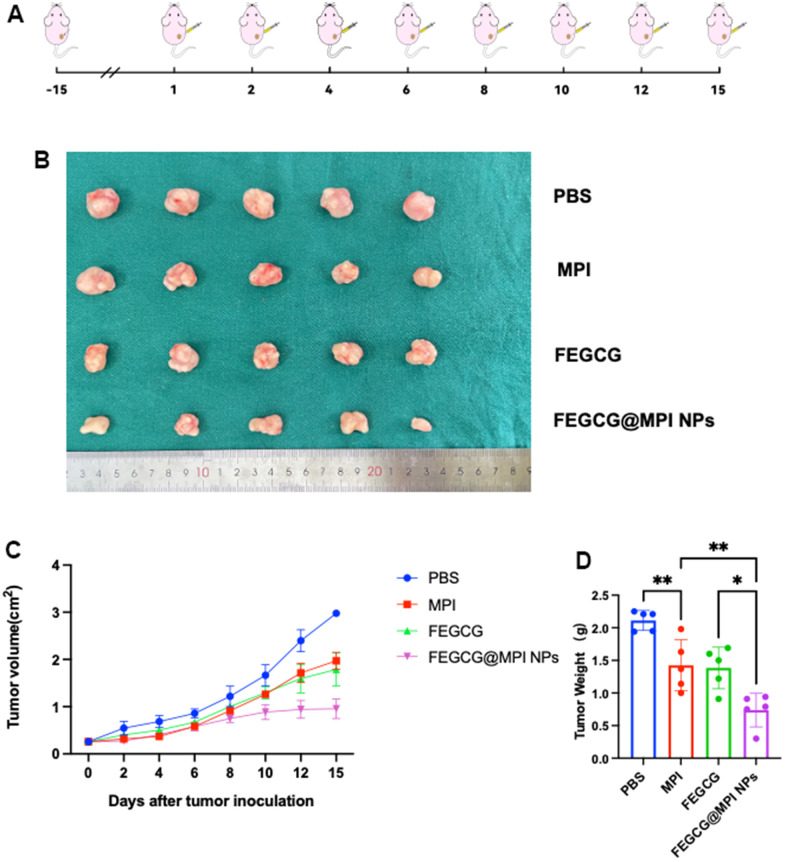
**Antitumor efficacy of FEGCG@MPI NPs *in vivo*.** (**A**) Experimental scheme. Hep3B cells (2× 10^6^) injected subcutaneously into nude mice. After two weeks, the mice were randomized and given the drug. (**B**) Representative photographs of collected tumor tissues. (**C**) The tumor growth curves of individual mice, and (**D**) the tumor weight of mice after treated with PBS, FEGCG, MPI, and FEGCG@MPI NPs (n=5). Data are presented as the means ± SD. Error bars represent the standard deviations of at least three separate measurements. *P < 0.05, **P < 0.01 by one-way ANOVA analysis followed by Turkey’s multiple comparisons.

Next, the *in vivo* efficacy was examined, and the treatment regime was showed in (Figure 5A). When the tumor model was established, different drugs were injected into tumor on 1st, 2nd, 4th, 6th, 8th, 10th, 12th, and 15th days. On the 16th day, all mice were killed for tumor weight and tumor volume (Figure 5B). The growth of tumor volume and weight of FEGCG/MPI NPs in the treatment group was significantly slower than that of the other three groups (Figure 5C, 5D). The results of immunohistochemistry are consistent with the *in vitro* experiments. The content of PD-L1 in control group was relatively high, and FEGCG@MPI NPs was relatively low (Supplementary Figure 2). These results indicated the superior antitumor efficacy of FEGCG@MPI NPs. The flow chart of our article is summarized in Supplementary Figure 3.

## DISCUSSION

The main purpose of this chapter is to improve the anti-tumor effect of FEGCG and MPI after packaging, while reducing the hemolytic side effects of MPI. In this study, experiments *in vitro* and *in vivo* have proved that FEGCG@MPI NPs have great potential to enhance the ability to kill Hep3B cells of liver cancer. At present, there are many different effective methods for the treatment of liver cancer at home and abroad. However, considering the stage of HCC diagnosis, it is necessary to see different treatment methods suitable for the patient and determine the best treatment plan. It is the most important to consider the patient’s burden and tolerance of the treatment plan, as well as the patient’s current status, potential liver function, liver accident disease and related complications. Although there are many difficulties, there are many corresponding treatment options: radiofrequency ablation, microwave ablation, hepatectomy, and liver transplantation, which are all potential options for the treatment of liver cancer [24]. In the past few decades, the application of injection drug delivery system in cancer treatment has made great progress. These improvements include liposomes, lipid nanoparticles (LNPs) and other nanoparticles with or without macromolecular conjugates. These drugs have the advantages of biocompatibility, controlled and sustained release of anti-tumor drugs and low toxicity. Valuable experiments on these drug delivery systems can better treat multidrug resistant cancer and reduce cardiac toxicity. At present, there are many related therapeutic schemes in the clinical and preclinical development of immunotherapy. Immunotherapy has revolutionized the clinical management of patients with different types of cancer by making autologous or allogeneic immune cells sensitive to the tumor microenvironment and ultimately leading to tumor cell lysis without killing normal cells quickly. Although immunotherapy is widely used at present, it is difficult to activate the immune system, and few cancer patients can respond to treatment. In addition, during and after treatment, there will be serious immune-related adverse reactions, and the emergence of nanoparticles can better solve this problem. It has been proposed in the literature that the latest progress in the development of lipid nanoparticles (NP) can not only deliver small molecules, but also deliver mRNA to achieve systemic anti-cancer immunity through cytotoxic immune cell activation, checkpoint blocking and chimeric antigen receptor cell therapy [25]. The rise of nanotechnology has led to the development of drug delivery systems that selectively target tumor-specific tissues [26]. Nanocarriers can regulate the pharmacokinetics and pharmacodynamics of drugs, thus improving the therapeutic index of drugs. By changing the biological transformation of drugs, it makes drugs accumulate, especially at tumor sites. Today, nanotechnology has been extended to the joint delivery of two or more drugs [27]. As expected by the design, melittin is a new drug for the treatment of liver cancer and a polypeptide extracted from bee venom. The molecular chemical formula C131H229N39O31, which accounts for about 50% of the dry weight of bee venom, is mainly composed of 26 amino acids. Melittin has antibacterial, antiviral, anti-inflammatory and anti-radiation effects. It has been studied in the field of treating liver cancer, breast cancer and stomach cancer. Melittin is a kind of amphoteric characteristic, N-terminal hydrophobicity, C-terminal hydrophilicity, which is exactly the amphoteric characteristic. MPI can better exist in cancer environment. MPI can directly dissolve the cell membrane and cause cell death, but it can also directly kill normal cells, which is also a hemolytic side effect that MPI cannot ignore. Previous studies have shown that melittin can inhibit the proliferation of HepG2 cells and induce cell death “1”. Melittin can activate the apoptosis pathway, down-regulate the ratio of Bcl-2/Bax, and then induce the apoptosis of Hep3B cells. At the same time, FEGCG is a substance extracted from tea polyphenols. It also has antiviral, anti-inflammatory and anti-tumor effects, which can inhibit the growth of various types of cancer cells and induce their apoptosis. FEGCG can inhibit the expression of PD-L1 induced by interferon and reduce the expression of STAT-1, P-STAT-1, IRF and PD-L1 through transcription level, thus inhibiting the growth of tumor cells. In this paper, FEGCG and MPI are combined into nanoparticles, and the MPI is wrapped with FEGCG, in order to enhance the anti-tumor effect of FEGCG and reduce the side effects of MPI. *In vivo* experiments included the establishment of subcutaneous tumor models in nude mice to verify the effect of FEGCG@MPI NPs on tumor. *In vitro* experiments include particle size measurement, delivery efficiency, CCK8 test, apoptosis test, cell uptake, Western blot, cell scratches, cell invasion, flow cytometry, confocal, cell hemolysis, immunohistochemistry, etc. CCK8 experiment showed the most suitable concentration of FEGCG and MPI, and finally the best concentration of the combination of the two drugs was obtained. Finally, it was verified that the combined drug was more effective than the single drug. The novel point of the experiment in this chapter is to propose a new idea: FEGCG wraps MPI to form nanoparticles to reduce the side effects of MPI hemolysis. It is speculated that the effect of nanoparticle encapsulation weakens the effect of MPI on destroying cell membrane.

## CONCLUSIONS

In summary, we have described a novel strategy to promote MPI-based cancer immunotherapy by constructing a fluorinated EGCG-based delivery system. The developed FEGCG@MPI NPs are formed through hydrogen-bond interactions between EGCG and MPI with improved MPI delivery efficiency. Then, the release of FEGCG and MPI can not only regulate PD-L1 signaling with IRF, STAT-1/pSTAT-1, and PD-L1 expression, but also induce apoptosis signaling with Bcl-2 and Bax expression in tumor cells. In addition, the combination of FEGCG and MPI decreases the hemolytic activity of MPI. The results presented in this study demonstrate that EGCG with fluorination can play the ideal carrier for MPI-based delivery and therapeutics, which represents a potential platform and promising strategy for enhanced anti-tumor immunotherapy.

## MATERIALS AND METHODS

### Chemicals and reagents

(−)-epigallocatechin-3-O-gallate (EGCG) was purchased from Energy Chemical (Shanghai, China). 2,2,3,3,4,4,5,5,6,6,7,7-dodecafluoro-1,8-octanediol (12F) were purchased from Macklin Biochemical Co. Ltd. (Shanghai, China). RIPA lysis buffers and Phenylmethanesulfonyl fluoride (PMSF) were obtained from Solarbio (China). The primary antibodies to Bax, Bcl-2, PD-L1, STAT-1, P-STAT-1, IRF and β-actin were purchased from Cell Signaling Technology (United States). The secondary antibodies to HRP AffinPure goat anti-rabbit IgG and HRP AffinPure goat anti-mouse IgG were purchased from Proteintech (United States). The Alexa Fluor®488 goat anti-mouse/rabbit IgG and Alexa Fluor®568 goat anti-mouse/rabbit IgG were purchased from Invitrogen (United States). PBS, DMEM, and EDTA were purchased from WISENT. Triton X-100 and 4, 6-dimethyl-2-phenylindole (DAPI) were purchased from Abcam (United Kingdom). Human hepatocellular carcinomas (Hep3B) were obtained from American type culture collection (ATCC). All other reagents were obtained from Beyotime Biotechnology Co., Ltd. (Shanghai, China) unless otherwise stated. Experimental data were obtained from Beiyu Biotechnology Co., Ltd. (Shanghai, China).

### Synthesis, preparation, and characterization of FECGC and FEGCG@MPI NPs

The fluoropolymer of FEGCG was synthesized through the reaction of fluorinated compound and EGCG with the ROS-sensitive linker of oxalyl chloride. FEGCG and MPI are combined by a hydrogen bond. The complexes were prepared by complexing the carrier with FEGCG@MPI NPs at w/w = 1 for 30 mins (FEGCG@MPI NPs concentrations = 10 μg mL^−1^). The particle size and zeta potential of FEGCG@MPI NPs complexes were determined by DLS. The loading efficiency of FEGCG@MPI NPs was determined using a FITC fluorescence quenching assay (excitation at 450 nm, emission at 480–600 nm).

### Cell toxicity, apoptosis and cellular uptake

Fresh blood samples from mice were placed in EDTA-containing tube. The blood was centrifuged at 3000 rpm at 4° C for 10 minutes to collect red blood cells (RBC), and then divided into five groups, which were: (i) PBS, (ii) MPI, (iii) FEGCG, (iv) FEGCG@MPI NPs, (v) Triton. After incubating these samples at 37° C for 60 minutes, the blood samples were centrifuged at 3000 rpm for 5 minutes to get the supernatant and the concentration of hemoglobin was measured at 545 nm. Data (means ± SD) were from three independent experiments.

Human Hepatoma cells were cultured in DMEM culture medium at 37° C with 5% CO2 atmosphere.

For testing the proliferative activity of cells, 2x10^3^ Hep3B cells were seeded into 96-well plate per well in 200 μl DMEM medium. On the second day, the cells were treated with various concentrations (10, 20, 50, 100, and 200μg/ml) of FEGCG and MPI (4, 6, 8, 12, and 24μg/ml) for 24 hours. Subsequently, the previous medium was removed, and 100 μl DMEM medium containing 10 μl Cell Counting Kit (CCK)-8 solution (Vazyme) were added to each well. The plate was incubated for 1 h, and the absorbance values per well were recorded at 450 nm. The resulting data were analyzed from three independent experiments and then normalized to the absorbance of the wells containing culture medium alone.

Hep3B cells (5×10^5^ cells) were seeded in six-well plates and incubated with PBS, MPI, FEGCG, and FEGCG@MPI NPs separately for 24h. Then the cells were digested with trypsin without EDTA and washed with PBS twice, suspended in binding buffer and stained with Annexin V-FITC/PI Apoptosis Detection Kit (Vazyme). The cells were cultured in darkness (37° C) for 20 minutes and apoptotic cells were observed using FACSC calibur flow cytometry (Sakuri, USA) within 1 h.

For cell delivery, Hep3B cells were cultured for confocal imaging or flow cytometry measurement. For EGCG delivery, cells were incubated with free MPI for 4 hours. For peptide delivery, cells were incubated with EGCG/MPI-FITC and FEGCG/MPI-FIT for 4 hours. After incubation, cells were washed with PBS, and DAPI was stained before confocal microscope, and cells were collected by flow cytometry.

### Cell migration and invasion

Briefly, Hep3B cells were seeded into 6-well plates containing complete growth media with 10% FBS. When the 98%-100% of the tissue culture dished was occupied by cells, scratch with the sterile 10-200 μl pipette tips was done. The scratch-wound healing cum cell migration was monitored over time and images were captured under a light microscope with 10× objective lens at the 0, 12, and 24 h time-points after denudation, and then images were analyzed with the ImageJ software.

For an invasion assay, 2 × 10^5^ Hep3B cells were seeded into 100 μL serum-free DMEM medium in the upper surface and allowed to invade toward the underside of the membrane in 24-well tissue culture plates containing 600 μL complete growth media with 10% FBS for 24 h. Then PBS, FEGCG, MPI, and FEGCG@MPI NPs were added to lower chamber for 24h. Finally, the invaded cells on the underside of the membrane were fixed with 4% methanol, and then stained with crystal violet dye for 30 minutes. After that, the sample is observed under an optical microscope.

### Proteins of PD-L1 and apoptosis pathway determination

Hep3B cells were lysed with Cell lysis buffer for Western and IP (Beyotime Biotechnology, Jiangsu, China) containing 1 % PMSF. The protein content of different fractions was detected via the bicinchoninic acid (BCA) method. The obtained protein samples were subjected to sodium dodecyl sulfonate polyacrylamide gel electrophoresis (SDS-PAGE) with 10% gel concentration, and transferred onto PVDF membranes with pre-cast gels using wet transfer with 250 mA for 1 h. Membranes were put in 5% skimmed milk and seal it for 2h, then the first antibody (anti-β-actin, 1:1000 PD-L1 (Abmart, Shanghai, China)), 1:1000 anti-STAT-1 (Abcam, MA, USA), anti-P-STAT1-1 (Abcam, USA), anti-IRF, Bcl-2 and Bad (Cell Signaling, USA) were incubated for 4° C overnight. The next day, membranes were probed with second antibody (HRP-anti-mouse/rabbit IgG Cat#7076, Cat#7074, antibody, CST, Beijing, China). After another three washes, signals were detected by LuminataTM Western HRP substrates (Millipore Corporation, Billerica, MA, USA) and Chemi-DocTM MP Imaging System (v5.2.1, Bio-Rad Laboratories, Inc, Hercules, CA, USA).

### Animals and tumor models

Athymic BALB/c nude mice (male, 6-8weeks) were purchased from the Gempharmatech Co., Ltd. All animal experiments were approved by the Institutional Animal Care and Use Committee of the Affiliated Drum Tower Hospital of Nanjing University Medical School (NO. 2018-289-01). For establishing a Hep3B tumor bearing mouse model, 2x10^6^ cells/0.2 mL PBS was subcutaneously implanted into the left armpit of the mice. When the tumor volume grew to approximated 200 mm^3^, the mice were randomly divided into four groups (*n* = 5) : (i) PBS, (ii) MPI, (iii) FEGCG, (iv) FEGCG@MPI NPs. The treatment was performed on days 1, 2, 4, 6, 8, 10, 12, 15 by intratumor injection, and the tumor volume and body weight of the mice were recorded every two days (Supplementary Figure 2). Tumor volume (mm^3^) = length × width^2^/2. On day 15, all mice were sacrificed and the tumors were collected for weight recording. After tumor resection, the tumor was weighed and sent to sections for immunohistochemical staining according to a standard procedure.

### Statistical analysis

Statistical analysis was performed by two-sided Student’s t-test for two groups and one-way ANOVA analysis of variance for multiple groups (when P < 0.05, the data were considered statistically significant).

### Availability of data and materials

The data used in the study are not public data. The datasets used in the study are available from the corresponding authors on reasonable requests.

## Supplementary Material

Supplementary Figures
