# Supplementary material for: Co-delivery of EGCG and melittin with self-assembled fluoro-nanoparticles for enhanced cancer therapy
Source: Aging (Albany NY). 2023 Jun 5;15(11):4875–88. doi: 10.18632/aging.204769 (PMC10292896; doi:10.18632/aging.204769)
Supplement: Supplementary Figures [file aging-15-204769-s001.pdf]

SUPPLEMENTARY FIGURES

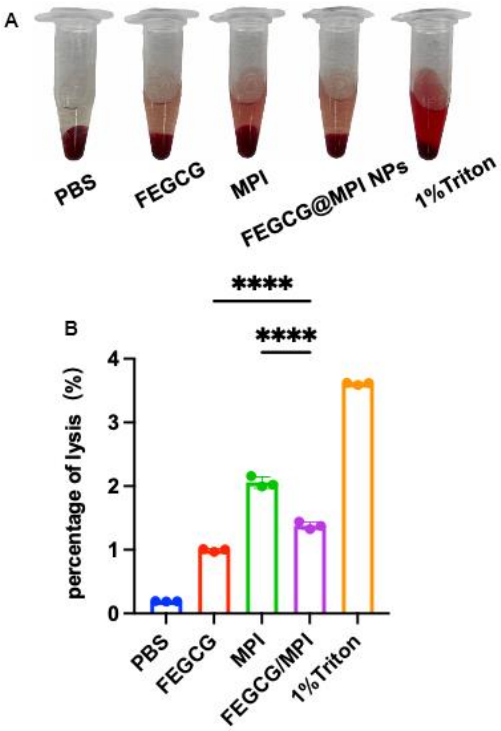

Supplementary Figure 1. (**A**) Hemolysis effect and quantitative lysis percentage (*n* = 3). (**B**) Data are presented as the mean ± standard deviation. *P < 0.05 vs EGCG/MPI group, **P < 0.01 vs EGCG/MPI group.

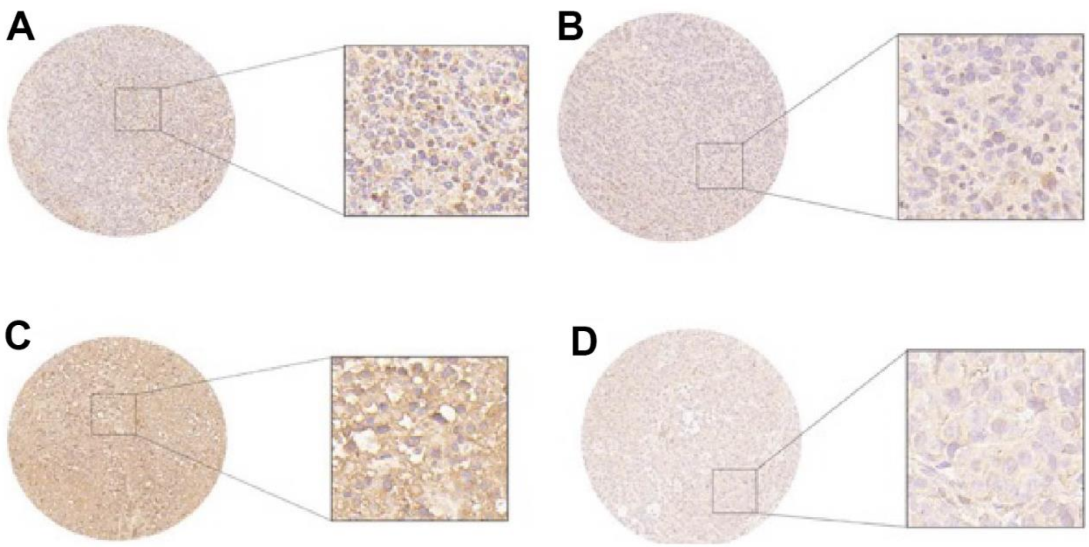

Supplementary Figure 2. Immunohistochemical staining (x 200) showed the expression of PD-L1 in tumor tissue. (**A**–**D**) tumor tissues treated with PBS, FEGCG, MPI, FEGCG@MPI NPs. The expression of PD-L1 was significantly decreased in FEGCG@MPI NPs group.

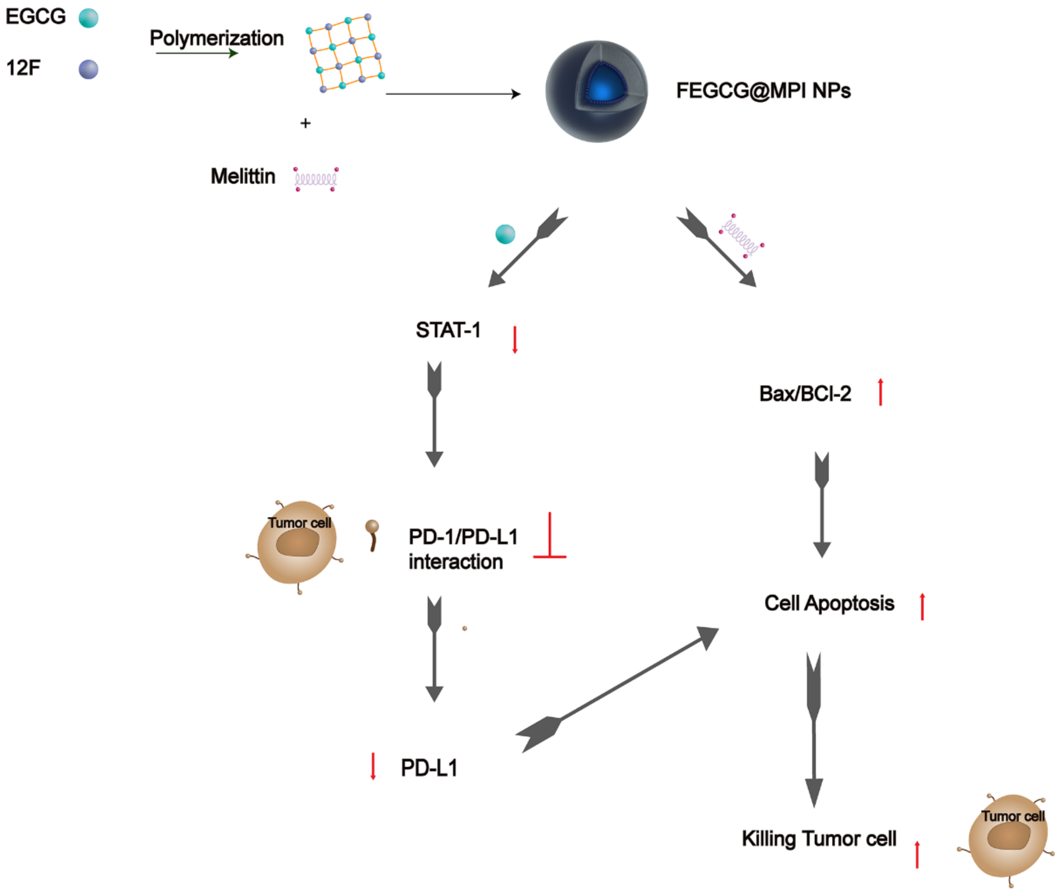

Supplementary Figure 3. Schematic diagram of FEGGC in collaboration with MPI in antitumor.
